# Ghrelin Receptor Antagonism of Methamphetamine-Induced Conditioned Place Preference and Intravenous Self-Administration in Rats

**DOI:** 10.3390/ijms19102925

**Published:** 2018-09-26

**Authors:** Tereza Havlickova, Chrysostomos Charalambous, Marek Lapka, Nina Puskina, Pavel Jerabek, Magdalena Sustkova-Fiserova

**Affiliations:** 1 Department of Pharmacology, Third Faculty of Medicine, Charles University, Ruska 87, 1010034 Prague, Czech Republic; terez.hav@gmail.com (T.H.); chrysostomos.charalambous@lf3.cuni.cz (C.C.); marek.lapka@centrum.cz (M.L.); pa.jerabek@gmail.com (P.J.); 2 Department of Addictology, First Faculty of Medicine, Charles University, Apolinarska 4, 212800 Prague, Czech Republic; nina.puskina@seznam.cz

**Keywords:** methamphetamine, ghrelin antagonism, conditioned place preference, intravenous self-administration, addiction, rat

## Abstract

Methamphetamine abuse imposes a significant burden on individuals and society worldwide, and an effective therapy of methamphetamine addiction would provide distinguished social benefits. Ghrelin significantly participates in reinforcing neurobiological mechanisms of stimulants, including amphetamines; thus, ghrelin antagonism is proposed as a promising addiction treatment. The aim of our study was to elucidate whether the pretreatment with growth hormone secretagogue receptor (GHS-R1A) antagonist, substance JMV2959, could reduce the methamphetamine intravenous self-administration (IVSA) and the tendency to relapse, and whether JMV2959 could reduce or prevent methamphetamine-induced conditioned place preference (CPP) in rats. Following an adequate maintenance period, JMV2959 3 mg/kg was administered intraperitoneally 20 min before three consequent daily 180 min sessions of methamphetamine IVSA under a fixed ratio FR1, which significantly reduced the number of active lever-pressings, the number of infusions, and the amount of the consumed methamphetamine dose. Pretreatment with JMV2959 also reduced or prevented relapse-like behavior tested in rats on the 12th day of the abstinence period. Pretreatment with JMV2959 significantly reduced the expression of methamphetamine-induced CPP. Simultaneous administration of JMV2959 with methamphetamine during the conditioning period significantly reduced the methamphetamine-CPP. Our results encourage further research of the ghrelin antagonism as a potential new pharmacological tool for methamphetamine addiction treatment.

## 1. Introduction

Methamphetamine is considered as a more addictive *N*-methylated derivate of amphetamine, developed in the 1950s [1,2]. Unlike the restricted medical use of amphetamine, methamphetamine is usually strictly illicit across the world. Serious health and social consequences linked with methamphetamine use are well documented [3]. Yet, according to the NIDA in the USA [4], about 6.5% of the population (26 years old and older) was assessed to have experienced methamphetamine in their lifetime and 0.5–0.3% have used methamphetamine during the last year and month respectively. The European Monitoring Centre for Drugs and Drug Addiction (EMCDDA) estimated 3.6% life-time prevalence and 0.5% last year use of amphetamines in Europe in 2016. Unlike cocaine, amphetamines are more prominent in northern and eastern European countries, with methamphetamine being common in the Czech Republic, Slovakia, Latvia, and Lithuania [5]. Methamphetamine injection use, which is prevalent in the Czech Republic, is the most devastating, and so far, symptomatic treatments for addiction remain insufficient. Therefore, new effective methamphetamine addiction treatment strategies are currently being intensively studied.

Recently, growth hormone secretagogue receptor (GHS-R1A) antagonism has been proposed to be a promising mechanism for drug addiction treatment (for references, see [6,7,8]. This addictological research approach is most advanced in the field of alcohol abuse. Already, a few controlled human studies recently confirmed that systemic administration of ghrelin significantly increased craving in heavy alcoholics [9,10], and a GHS-R1A inverse agonist is being tested in alcoholics within an initial clinical study [11]. However, it appears that ghrelin importantly participates also in other types of drug dependence. Several experimental studies have supported the role of ghrelin in the psychostimulant reward system. Rats that were injected intraperitoneally with ghrelin prior to cocaine exhibited increased cocaine-induced locomotor hyperactivity [12]. Also, repeated systemic administration of ghrelin and ghrelin application into the nucleus accumbens (NAC) potentiated cocaine-induced hyperlocomotion [13,14]. Pretreatment with systemic ghrelin and ghrelin microinjected into the ventral tegmental area (VTA) significantly augmented the rewarding effects of sub-threshold cocaine doses in rat conditioned place preference (CPP) [15,16]. Genomic and pharmacological ablation of GHS-R1A significantly reduced cocaine-induced locomotor stimulation and cocaine behavioral sensitization in mice [17,18,19]. The ghrelin antagonist JMV2959 attenuated cocaine-, nicotine- and amphetamine-induced locomotor stimulation, accumbens dopamine release, and CPP in mice [20,21] and nicotine-induced sensitization in rats [22]. In the dopamine-transporter knock-out mice, an acceptable model of extracellular dopamine overflow similar to amphetamine effects, GHS-R1A antagonist YIL781 significantly reduced the observed hyper-locomotion [23]. Subchronic GHS-R1A blockade with JMV2959 also attenuated the amphetamine-induced locomotor stimulation in mice [24].

Amphetamine and methamphetamine generally induce similar effects in animal models, such as similar drug intake in intravenous self-administration in rats [25] or monkeys [26]. However, several studies showed more potent effects of methamphetamine in neurochemical and behavioral studies [27,28]. The current literature is very limited so far concerning the methamphetamine–ghrelin system relationship. Kobeissy et al. [29] described that acute administration of 5, 20, and 40 mg/kg intreaperitoneal (i.p.) methamphetamine affected ghrelin serum levels in rats, which may participate in drug-induced hypophagia. One clinical genetic study [30] showed no association between pre-proghrelin gene (GHRL) variations, and susceptibility to the development of methamphetamine dependence in a sample of Korean population, but found a significant correlation between carrying the GHRL single nucleotide (Leu72Met) polymorphism and emotional problems, such as depression or anxiety, which are associated with drug addiction.

Thus, the research findings so far suggest that ghrelin importantly participates in reinforcing the neurobiological mechanisms of stimulants, including amphetamines. However, to our knowledge, the applicability of ghrelin antagonism for reduction of stimulant consumption in a self-administration model of addiction has never been tested. Therefore, our study aim determines whether pretreatment with GHS-R1A antagonist, substance JMV2959, could decrease methamphetamine intravenous self-administration (IVSA) and tendency to relapse, in rats. The drug-CPP model provides unique information about the rewarding effects of contextual cues associated with a drug experience, which play an important role in addiction [31]. Therefore, we test in rats whether ghrelin antagonism could prevent or reduce the process of methamphetamine–CPP and/or reduce the expression of methamphetamine-induced CPP.

## 2. Results

### 2.1. Conditioned Place Preference

**Figure 1:**
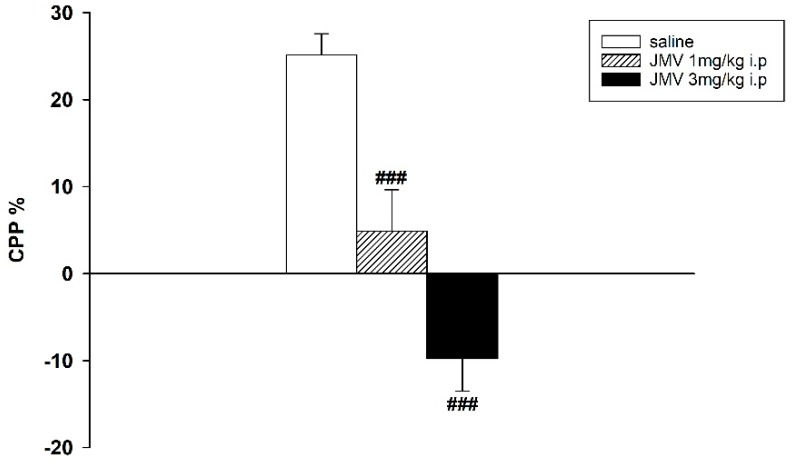
Effects of GHS-R1A antagonist on 2 mg/kg methamphetamine-induced conditioned place preference (CPP) in rats. After eight days of conditioning with 2 mg/kg i.p. methamphetamine, JMV2959 was administered in a single dose 20 min before testing (*N* = 8; means ± SEM). The effects of JMV2959 pretreatments in comparison to the saline group are expressed as ### *p* < 0.001.

**Figure 2:**
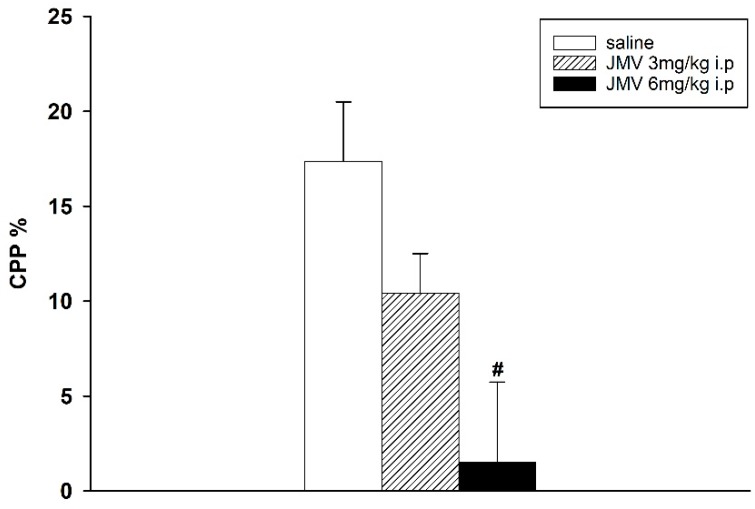
Effects of GHS-R1A antagonist on 5 mg/kg methamphetamine-induced CPP in rats. After eight days of conditioning with 5 mg/kg i.p. methamphetamine, JMV2959 was administered in a single dose 20 min before testing (*N* = 8; means ± SEM). Effects of JMV2959 pretreatments in comparison to the saline group are expressed as # *p* < 0.05.

**Figure 3:**
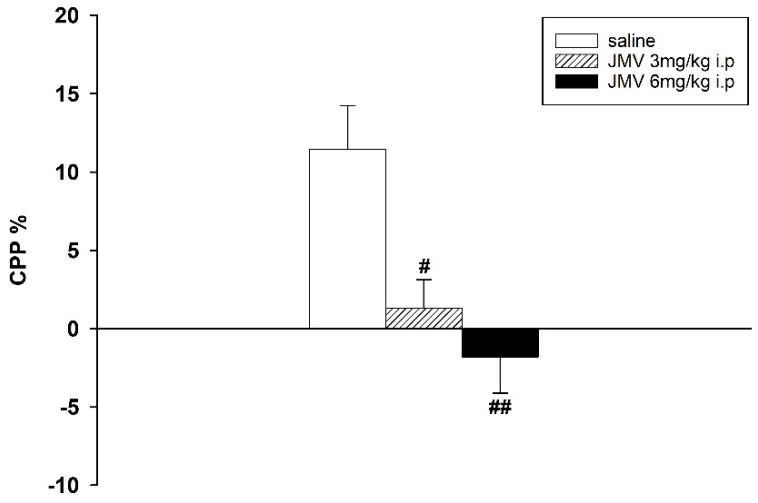
Effects of GHS-R1A antagonist on the conditioning procedure of 5 mg/kg methamphetamine-CPP in rats. During the eight days of conditioning, JMV2959 was administered repeatedly together with 5 mg/kg i.p. methamphetamine (*N* = 8 in the saline and *N* = 9 in the JMV2959 groups; means ± SEM). Effects of JMV2959 pretreatments in comparison to the saline group are expressed as # *p* < 0.05, ## *p* < 0.01.

Methamphetamine-induced CPP was manifested in both experimental designs. However, when the lower conditioning methamphetamine dose 2 mg/kg i.p. was used, the difference of percentage of the total time spent in the methamphetamine-paired compartment during the post-conditioning and the pre-conditioning session was higher than with 5 mg/kg; thus the lower methamphetamine dose seems more rewarding (see the Figure 1, Figure 2 and Figure 3).

#### 2.1.1. Antagonism of Methamphetamine-CPP Expression

When the GHS-R1A antagonist was administered in a single dose 20 min prior the test session on the post-conditioning day, the expression of the 2 as well as 5 mg/kg methamphetamine-CPP was significantly and dose-dependently reduced. The effects of single injections of 1 and 3 mg/kg JMV2959 administered after 2 mg/kg methamphetamine-CPP conditioning were highly significant: F2,21 = 29.38, *p* < 0.001 (see the Figure 1). Single doses of 3 and 6 mg/kg JMV2959 attenuated the 5 mg/kg methamphetamine-CPP with significance: F2,21 = 3.95, *p* = 0.036 (see the Figure 2).

#### 2.1.2. Antagonism of the Methamphetamine-CPP Conditioning Process

When JMV2959 3 and 6 mg/kg were administered repeatedly together with methamphetamine 5 mg/kg during conditioning, the methamphetamine-CPP was significantly reduced: F2,23 = 7.81; *p* = 0.003 (see Figure 3).

### 2.2. Intravenous Self-Administration

**Figure 4:**
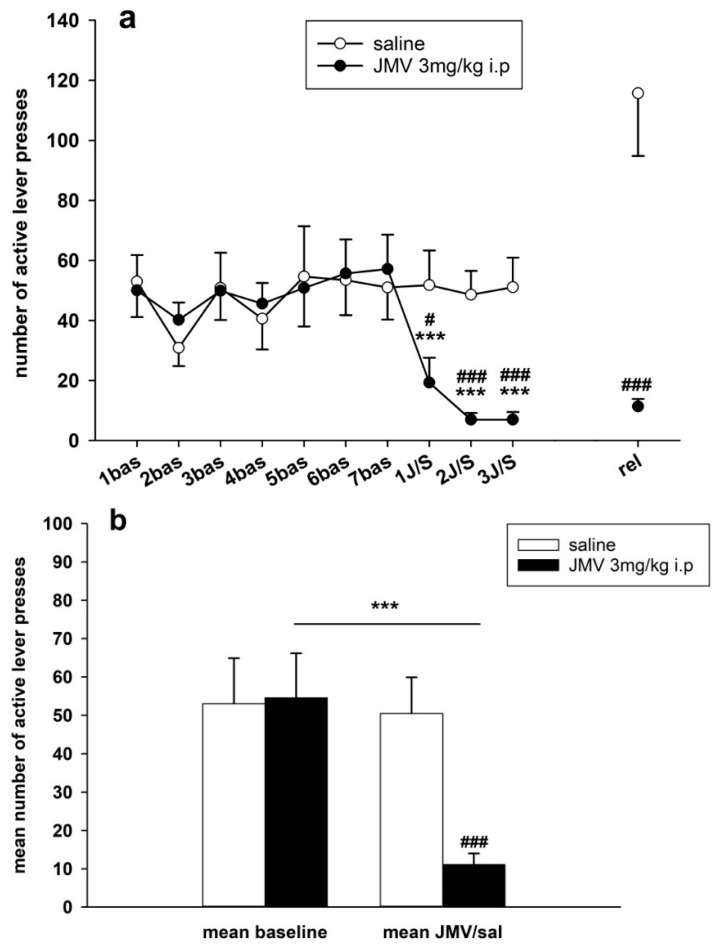
JMV2959 pretreatment effects on active lever-pressing during the methamphetamine intravenous self-administration (IVSA). JMV2959 3 mg/kg or saline were administered i.p. 20 min before the sessions. In the graph (**a**) active lever-pressings within daily 3 h sessions are illustrated during the last week before pretreatments, during the three pretreatment days and during the relapse-like behavior testing on the 12th day of abstinence, when the rats were not connected to the infusion pump. The mean of the last three baselines before the pretreatment (5–7 bas) was used for statistical analysis by two-way ANOVA for repeated measures (RM). Means of the rat groups are presented ± SEM; *N* = 12 (JMV2959 group), *N* = 8 (saline group). In the graph, (**b**) mean JMV2959/saline active lever-pressing (1–3 JMV/sal) are illustrated together with the mean baselines (5–7 bas). All of the data of the three last baselines and the three pretreatments were used for two-way ANOVA analysis. The effects are shown as follows: saline (open circle, open bar), JMV2959 (filled circle, filled bar). Differences between the groups are expressed as # *p* < 0.05, ### *p* < 0.001. Differences to baseline mean are expressed as *** *p* < 0.001, and the horizontal arrow shows the two appropriate bars.

**Figure 5:**
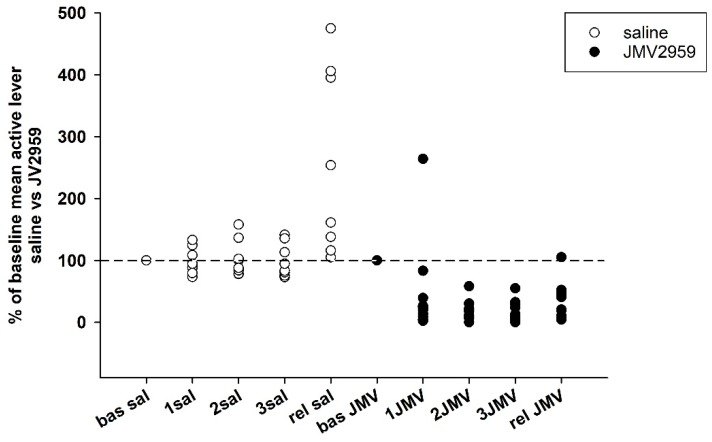
JMV2959 and saline effects on active lever-pressing during methamphetamine IVSA in single rats in percentage of baseline mean (mean of last three baselines before pretreatment). JMV2959 3 mg/kg and saline were administered i.p. 20 min before the session. The effects are illustrated as follows: saline (open circle), JMV2959 (filled circle). The dotted line shows the baseline mean level (100%; “bas”). During the relapse-like behavior testing session (“rel”), the rats were not connected to the infusion pump.

**Figure 6:**
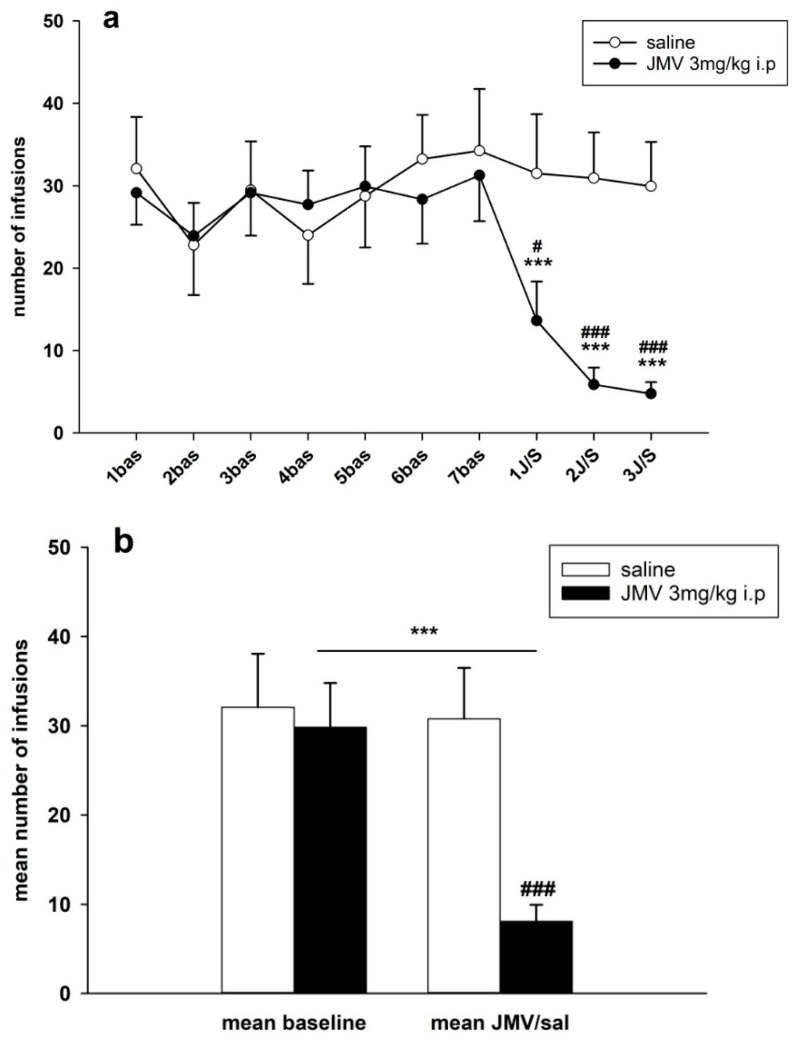
JMV2959 pretreatment effects on infusions number during the methamphetamine IVSA. JMV2959 3 mg/kg or saline were administered i.p. 20 min before the sessions. In the graph, (**a**) the number of infusions within daily 3 h sessions are illustrated during the last week before pretreatments and during the three pretreatment days. The mean of the last three baselines before the pretreatment (5–7 bas) was used for statistical analysis with two-way ANOVA RM. Means of the groups are presented ± SEM; *N* = 12 (JMV2959 group), *N* = 8 (saline group). In the graph (**b**) the mean JMV2959/saline effects (1–3 JMV/sal) are illustrated together with the mean baselines (5–7 bas); two-way ANOVA was used for statistical analysis. The effects are showed as follows: saline (open circle, open bar), JMV2959 (filled circle, filled bar). Differences between groups are expressed as # *p* < 0.05, ### *p* < 0.001. Differences to baseline mean are expressed as *** *p* < 0.001, the horizontal arrow shows the two appropriate bars.

**Figure 7:**
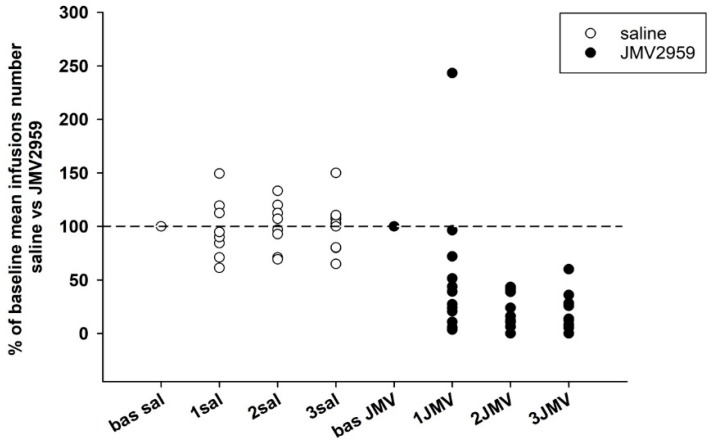
JMV2959 and saline effects on number of infusions during methamphetamine IVSA in single rats in percentage of baseline mean. JMV2959 3 mg/kg and saline were administered i.p. 20 min before the session. The effects are illustrated as follows: saline (open circle); JMV2959 (filled circle). The dotted line shows the baseline mean level (100%).

**Figure 8:**
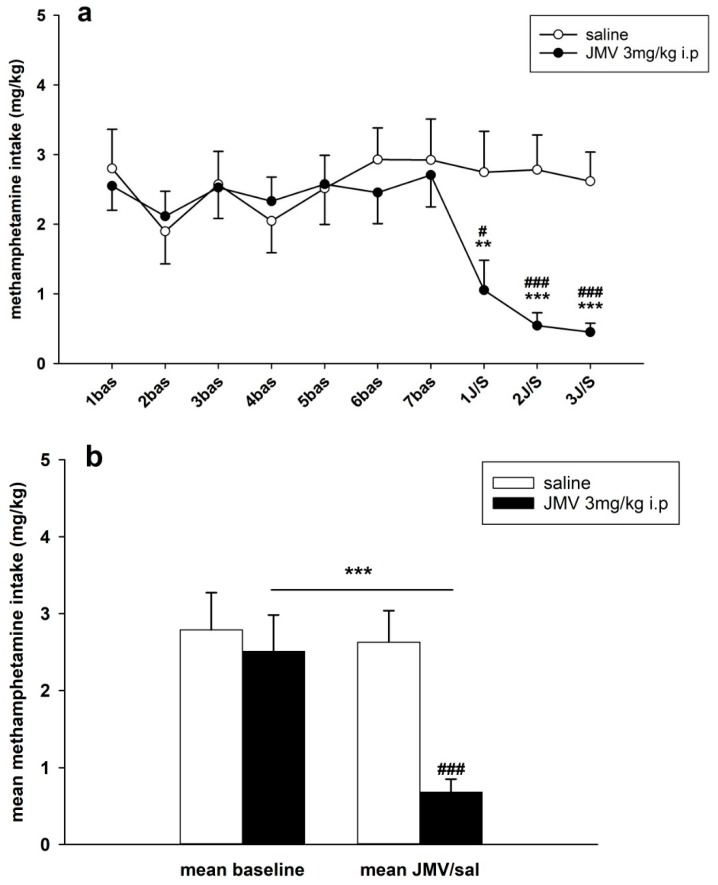
JMV2959 pretreatment effects on methamphetamine consumption/dose mg/kg during the IVSA. JMV2959 3 mg/kg or saline were administered i.p. 20 min before the sessions. In the graph, (**a**) methamphetamine intake mg/kg within daily 3 h sessions are illustrated during the last week before pretreatments and during the three pretreatment days. The mean of the last three baselines before the pretreatment (5–7 bas) was used for statistical analysis with two-way ANOVA RM. The means of the groups are presented ± SEM; *N* = 12 (JMV2959 group), *N* = 8 (saline group). In the graph (**b**) the mean JMV2959/saline effects (1–3 JMV/sal) are illustrated together with the mean baselines (5–7 bas); two-way ANOVA was used for statistical analysis. The effects are shown as follows: saline (open circle, open bar), JMV2959 (filled circle, filled bar). Differences between the groups are expressed as # *p* < 0.05, ### *p* < 0.001. Differences to baseline mean are expressed as ** *p* < 0.01, *** *p* < 0.001, the horizontal arrow shows the two appropriate bars.

**Figure 9:**
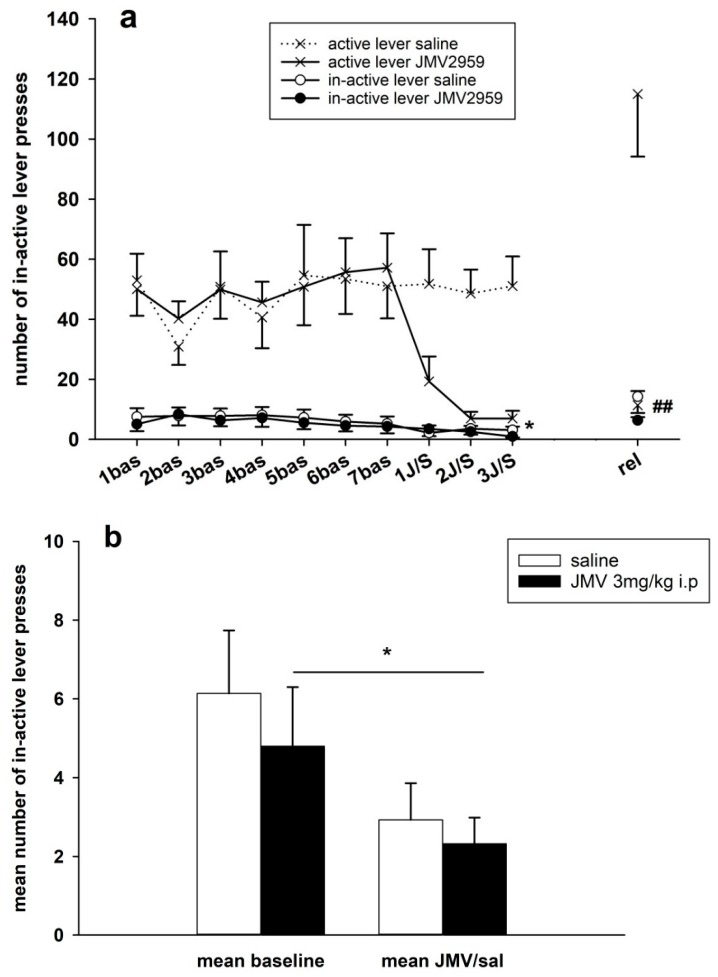
Changes of inactive lever-pressing during the methamphetamine IVSA and JMV2959 pretreatments. JMV2959 3 mg/kg or saline were administered i.p. 20 min before the sessions. In the graph (**a**) inactive lever-pressings within daily 3 h sessions are illustrated during the last week before pretreatments, during the three pretreatment days and during the relapse-like behavior testing on the 12th day of abstinence, when the rats were not connected to the infusion pump. Mean of the last three baselines before the pretreatment (5–7 bas) was used for statistical analysis by two-way ANOVA RM. Means of the rat groups are presented ± SEM; *N* = 12 (JMV2959 group), *N* = 8 (saline group). In the graph, (**b**) the mean JMV2959/saline inactive lever-pressing (1–3 JMV/sal) are illustrated together with the mean baselines (5–7 bas); two-way ANOVA was used for analysis. The effects are showed as follows: saline (open circle, open bar), JMV2959 (filled circle, filled bar). Differences between groups are expressed as ## *p* < 0.01. Differences to baseline mean are expressed as * *p* < 0.05, the horizontal arrow shows the two appropriate bars.

The last three daily 180 min sessions from a total of 14 sessions prior pretreatments were used as baseline data (*N* = 12 JMV2959 and 8 saline rats). The initial paired samples *t*-test comparing all baseline data revealed significant differences between active and inactive lever-pressing: *t* (118) = 10.297; *p* < 0.001. We observed distinct inter-individual differences in the basal methamphetamine consumption among all rats; however, the means of basal methamphetamine intravenous self-administration (the last three sessions prior to pretreatments) did not differ between the JMV2959 versus saline pretreated groups in all monitored parameters—number of active and inactive lever presses, number of infusions, and methamphetamine consumption (mg/kg) (see the Figure 4, Figure 5, Figure 6, Figure 7, Figure 8 and Figure 9).

#### 2.2.1. JMV2959 Pretreatment Effects on Methamphetamine IVSA

Methaphetamine self-administration was significantly reduced by pretreating with 3 mg/kg JMV2959 i.p. 20 min prior the IVSA session during all three consequent days of pretreatment in all monitored parameters, except for inactive lever-pressing, with antagonism being most greatly expressed on the second and third days (see Figure 4, Figure 5, Figure 6, Figure 7, Figure 8 and Figure 9).

Changes in active lever-pressing are illustrated in Figure 4a,b. The representative average basal active lever-pressing (mean of 5–7 baselines), which was used in the statistical analysis, was 53.0 ± 11.9 in the saline group and 54.5 ± 11.6 in the JMV2959 group. A two-way ANOVA for repeated measures (RM) followed by Bonferroni’s test revealed a significant decrease of active lever-pressing during the 3 h test sessions whenever JMV2959 was administered 20 min before the session, in comparison to the saline group (F1,18 = 9.68, *p* = 0.006), with a significant effect from the time/procedure (F3,54 = 6.80, *p* < 0.001); also, the time course of active lever presses during the sessions after pretreatment differed significantly between the two rat groups (F3,54 = 6.30, *p* < 0.01). The JMV2959 antagonism was obviously more pronounced on the second and third days. During the first day of JMV2959 pretreatment, we observed differences in individual reactivity of the rats—see Figure 5. Ten out of the 12 rats reduced active lever pressing to below 40% of the baseline mean (2–39%), one rat did not differ from the baseline, and one rat increased active lever-pressing to 264% of baseline mean. However, on the second and third pretreatment days/sessions, these two rats reduced active lever-pressing to 11% plus 3% (first rat) and 10% plus 5% (second rat), respectively. We believe, that these observed differences represent inter-individually different reactions of the subjects to the suddenly dropped/lost reward in a previously learned/conditioned and reliably working procedure (see the Discussion). In the saline group (*N* = 8), active lever-pressing during the first pretreatment day lay within 73% and 132% of the baseline mean. The Figure 4b illustrates the general effect of JMV2959/saline administration (data of three pretreatment days: 1–3 JMV/sal) on active lever-pressing in methamphetamine IVSA (data of 5–7 baselines). The two-way ANOVA followed by Bonferroni’s test revealed significant differences between the rat groups (F1,1 = 15.48, *p* < 0.001), the effect of JMV2959 pretreatment/procedure (F1,116 = 10.48, *p* < 0.01) and group × pretreatment effects (F1,116 = 12.25, *p* < 0.01).

Changes in the number of methamphetamine infusions obtained within daily sessions are illustrated in Figure 6a. In our IVSA procedure, a 15 s time-out was used following each administered infusion, when the active lever-pressing was not rewarded with the infusion. The average basal infusions number (mean of 5–7 baselines), used in the statistical analysis, was 32.1 ± 6.0 in the saline group and 29.8 ± 4.9 in the JMV2959 group. In accordance with the active lever-pressing results, JMV2959 reduced significantly the number of infusions. A two-way ANOVA RM revealed significant difference between groups (F1,18 = 11.33, *p* = 0.003), a significant effect of time/sessions (F3,54 = 6.74, *p* < 0.001); time/session × group interaction (F3,54 = 5.12, *p* < 0.01). On the second and third days of pretreatment, JMV2959 antagonism was more pronounced and similar to the active lever-pressing during the first pretreatment day, and we observed distinct inter-individual differences in the reactions to JMV2959 administration among the rats—see Figure 7. On the first day of JMV pretreatment within 10 rats, the number of infusions was reduced to below 72% of the baseline mean (4–72%); one rat did not differ from baseline and one rat increased an infusion number of 243% of baseline mean. Again, these rats reduced the number of infusions on the second and third days; one rat to 16% plus 8%, and the second rat to 11% plus 5%, respectively. In the saline group, the infusion numbers were found to be within 64% and 149% of the baseline mean during the first pretreatment day. The two-way ANOVA followed by Bonferroni’s test was used for the evaluation of the general JMV2959 effect on number of infusions during the methamphetamine IVSA, and it revealed significant differences between the rat groups (F1,1 = 15.48, *p* < 0.001), a significant effect of the JMV2959 pretreatment/procedure (F1,116 = 10.48, *p* < 0.01) and group × pretreatment effect (F1,116 = 12.25, *p* < 0.01); see Figure 6b with the infusion means of baselines and the means of the pretreatments.

The results of methamphetamine daily consumption/dose mg/kg analysis are illustrated in the Figure 8a. In our IVSA study 0.09 mg/kg/infusion of methamphetamine was used. The average basal methamphetamine daily consumed doses, which were used in the statistical evaluation, were 2.8 ± 0.5 mg/kg (saline group) and 2.6 ± 0.4 mg/kg (JMV2959 group). Two-way ANOVA RM followed by Bonferroni’s test revealed significant differences between groups (F1,18 = 12.28, *p* = 0.003), effect of time/session (F3,54 = 6.75, *p* < 0.001) and time/session × group interaction (F3,54 = 5.66, *p* < 0.01). The inter-individual variability of the daily doses consumed by the rats after the JMV2959 pretreatments was very similar to the infusions. A Comparison of average the methamphetamine intake during the three baselines and the three pretreatment days are illustrated in Figure 8b; a two-way ANOVA analysis of all of the involved data confirmed a significant difference between JMV2959 and saline groups (F1,1 = 16.13, *p* < 0.001), the effect of pretreatment (F1,116 = 19.05, *p* < 0.001), and the group vs. pretreatment effect (F1,116 = 13.83, *p* < 0.001).

Changes in the inactive lever-pressing during the last week before pretreatment, during the pretreatment days and on the relapse-like behavior testing session, when the rats were not connected with the pump, are demonstrated in Figure 9a. Together with indication of active lever-pressing, thus the difference between active and inactive lever-pressing is presented. The data near zero were again inter-individually variable. The average basal inactive lever-pressing, used in the statistic, were 6.1 ± 1.6 in the saline and 4.8 ± 1.5 in the JMV2959 group. The two-way ANOVA RM found no significant difference between the groups (F1,18 = 0.76, n.s.), a significant effect of time/procedure (F3,54 = 3.53, *p* < 0.05), and no significant effect of group vs. time/procedure (F3,54 = 0.90, n.s.); Bonferroni’s test detected only a significant decrease in inactive lever-pressing in comparison to the baseline mean on the third day of pretreatment within the JMV2959 group (*p* < 0.05). The three days baseline means and the means of three days with the JMV/saline pretreatments are illustrated in Figure 9b. The two-way ANOVA confirmed no statistical significance between the groups (F1,1 = 0.94, n.s.), a significant effect of pretreatment/procedure (F1,116 = 8.08, *p* < 0.01), and no significant group vs. procedure effects (F1,116 = 0.13, n.s.).

#### 2.2.2. JMV2959 Pretreatment Effects on Methamphetamine Relapse-Like Behavior

**Figure 10:**
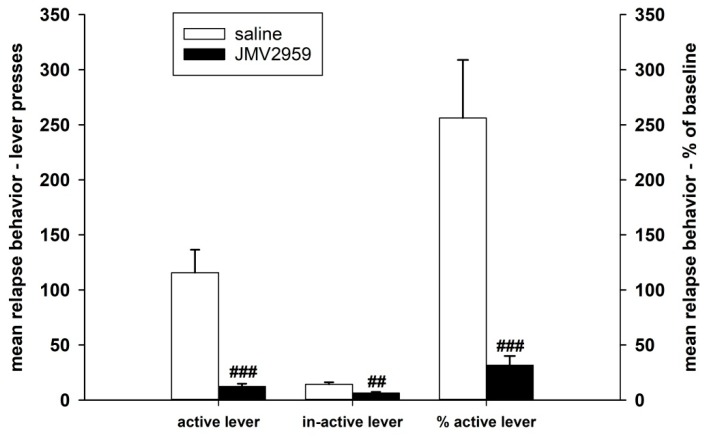
JMV2959 pretreatment effects on lever-pressing relapse-like behavior, observed after the 10–12th day of abstinence of the methamphetamine IVSA. JMV2959 3 mg/kg or saline were administered i.p. 20 min before the session. Means of the groups are presented ± SEM; *N* = 12 (JMV2959 group), *N* = 8 (saline group); Kruskal–Vallis ANOVA was used for the statistical analysis of the absolute data or the percentage of baseline means. The effects are shown as follows: saline (open bar), JMV2959 (filled bar). Differences between groups are expressed as ## *p* < 0.01, ### *p* < 0.001.

After 10–12 days of abstinence, when the rats were single housed in home cages, they were put back to their IVSA cages under the usual conditions, which were not only connected to the infusion pumps; thus the active lever-pressing were not rewarded. Twenty minutes before this session again to the appropriate rats were i.p. administered JMV2959 3 mg/kg or saline; the relapse-like behaviors of the rats was again tested for 180 min—see Figure 10. The average relapse-like active lever-pressing were 115.7 ± 20.9 in the saline and 12.3 ± 2.5 in the JMV2959 group, which were in percentage of baseline mean 256.2 ± 52.6% and 31.7 ± 8.3% respectively. The Kruskal–Vallis ANOVA revealed significant difference between groups—H(1) = 12.15, *p* < 0.001 using absolute values or H(1) = 13.15, *p* < 0.001 using percentage of baseline respectively. Within the saline group, we observed distinct differences in the inter-individual reactions of rats to this situation, as is illustrated in Figure 4 and Figure 5. Two saline rats pressed the active lever at a similar level to the baseline mean, two rats showed a moderate increase of 137–160% of the baseline mean, but this was still in the ranks of the saline pretreatment period, and half of a total of eight rats were pressing the active lever from 2.5 times to 4.7 times more frequently in comparison to the baseline mean (254–475%). Within the JMV2959 group, one rat pressed the active lever similarly to the baseline mean, and in all of the other 11 rats, active lever-pressing was reduced to a minimum of below 52% of the baseline mean (7–52%). Thus, administration of JMV2959 before the relapse session and possibly also as a consequence of the three pretreatments with JMV2959 during the last three sessions before abstinence were linked with a reduced/missing reward, and reduced relapse-like behavior in the rats, or at least prevented the increase of the active lever-pressing observed in the saline rats. Within the inactive lever-pressing during the relapse-like behavior testing, we also observed a significant reduction of pressing in the rats administered with JMV2959 in comparison to the saline group H(1) = 8.73 (Kruskal–Vallis ANOVA). However, the inactive lever pressing was rather low, with an average of 14.2 ± 1.9 in saline, and 6.3 ± 1.1 inactive lever-presses, and because the baseline mean was zero in some rats, it was not possible to calculate the percentage of the baseline mean for the inactive lever-pressing.

The changes in the body mass of the rats within the IVSA study were evaluated during the chosen periods and are summarized in the Table 1. The body mass changes were calculated within the last eight days before pretreatment, during the three days of pretreatment, the day of relapse-like behavior testing, and during all evaluated periods together (8 baselines + 3 pretreatment days + relapse-like behavior day = total 12 days). A two-way ANOVA RM followed by Bonferroni’s test documented no significant difference of rat body weights between the two groups—JMV2959 vs. saline group. No significant impact of three days of JMV2959 administration on the body mass of the rats was detected. In both rat groups, we observed a significant gain of weight at the end of the 12-days abstinence period.

## 3. Discussion

The presented results showed to our knowledge that for the first time, ghrelin antagonism significantly reduced methamphetamine intravenous self-administration and relapse-like behavior in rats. Further, we have found that the ghrelin antagonist significantly reduced methamphetamine-induced CPP expression, and simultaneous repeated administration of JMV2959 together with methamphetamine during conditioning also decreased the development of methamphetamine-CPP in rats.

Intravenous self-administration (IVSA) and drug-CPP represent crucial experimental models for the investigation of addictive properties and mechanisms, as well as the testing of potential new treatment approaches and medicines [31,32]. IVSA is able to estimate drug rewarding and reinforcing abilities and to evaluate the principle treatment goal of reducing or abolishing drug-taking behavior. CPP, where the drug of abuse is administered by the experimenter, measures drug reward and reinforcing properties (indirectly), and it is mainly focused on the association and conditioning of environmental cues with the drug effect, which plays an important role in the acquisition and maintenance of addiction. Despite prominent concordance between drugs, especially in rat models, IVSA processes are mediated, at least in part, by a neuropharmacological circuitry distinct form that subserves CPP [33,34].

The central ghrelin secretagogue receptors (GHS-R1A) are located on neurons within VTA, striatum, NAC, hippocampus, and the prefrontal cortex, and further important reward-related areas [35,36,37,38]. Ghrelin participates significantly in the rewarding properties of psychostimulants (for references see [6,7,19]), most likely through the modulation of the mesolimbic dopamine system in cooperation with the glutamate and acetylcholine systems [39,40]. Beside other effects, a ghrelin antagonist (JMV2959 i.p.) significantly attenuated cocaine, nicotine, and amphetamine-induced increases in accumbens dopamine release in mice [20,21]. Thus, presumably, cutting off of methamphetamine-rewarding abilities played a crucial role in the observed decrease of CPP development when a ghrelin antagonist (3 and 6 mg/kg JMV2959 i.p.) was injected together with methamphetamine (5 mg/kg i.p.) during conditioning. In our study, both methamphetamine-conditioned doses of 2 and 5 mg/kg i.p. induced biased CPP in Wistar male rats, although a lower methamphetamine dose 2 mg/kg produced higher CPP expression in comparison to 5 mg/kg, which suggests higher rewarding properties that are linked with the lower dose. This confirms literary search-findings where CPP was significantly expressed by systemic methamphetamine doses ranging between 0.125–5 mg/kg, and the most rewarding doses lying between 0.5–2.5 mg/kg i.p. [41,42,43]. Higher methamphetamine doses (starting around 5 mg/kg) are frequently injected in humans (regardless of tolerance), producing nearly hallucinogenic/psychotic effects. Pretreatment with JMV2959 also significantly and dose-dependently reduced the CPP expression of both conditioned methamphetamine doses (2 and 5 mg/kg); however, the antagonistic JMV2959 effect was more pronounced in rats with higher CPP expression (2 mg/kg methamphetamine conditioned rats), when 1 and 3 mg/kg JMV2959 doses reduced the CPP expression with high significance. Within the 5 mg/kg methamphetamine conditioned rats, a significant decrease of CPP expression was observed with a rather high 6 mg/kg JMV2959 dose, and the 3 mg/kg JMV2959-induced decrease did not reach significance. Thus, JMV2959 pretreatment significantly reduced the manifestation of developed place-conditioning with methamphetamine experiences, suggesting that ghrelin antagonism decreased cravings and the anticipation of a previously imprinted reward. It was previously demonstrated in mice that JMV2959 pretreatment reduced nicotine, cocaine, and dex-amphetamine-induced CPP expression [20,21], and in rats, systemic ghrelin-augmented cocaine CPP [15], which is, in principle, in accordance with our results. It was also confirmed that JMV2959 alone did not induce conditioned place preference in mice [44], and 3 mg/kg i.p. JMV2959 did not produce conditioned taste aversion in rats [45]. We have previously described that the administration of 3 mg/kg i.p. JMV2959 did not significantly influence the accumbens dopamine in rats, and 1, 3, and 6 mg/kg i.p. JMV2959 doses did not significantly influence rat locomotor activity [46,47].

Similarly to natural reinforcers (palatable food etc.), drugs of abuse can control the behavior of laboratory animals in an operant paradigm with a high correlation to humans [32]. Our methamphetamine self-administration study in rats seemed during the maintenance period to be roughly in accordance with the literature considering the IVSA behavior, the consumed dose, and also the inter-individual variability of the data [23,48,49,50]. Food deprivation, a state associated with increased ghrelin blood levels, augmented the self-administration of amphetamine and cocaine in rats [51,52]. In our study, pretreatment with GHS-R1A antagonist for three consecutive days significantly reduced the maintained methamphetamine IVSA in rats, using FR1, suggesting that JMV2959 notably reduced the reinforcing qualities of the self-administered methamphetamine. Ten of the 12 rats significantly reduced the methamphetamine IVSA already on the first JMV2959 pretreatment day, indicating that these rats recognized and accepted that the active lever-pressing was no longer coupled with any substantial reward. One rat surprisingly increased active lever-pressing dramatically during the first JMV2959 pretreatment day without any apparent change of inactive lever-pressing, and in the same rat we observed a significant IVSA decrease only on the second and third pretreatment days. We can speculate that this particular rat responded to the sudden lack of expected reward with increased effort to get it. According to the literature, similar observations can be found for the first day during the extinction period, when the active lever-pressing is often higher in comparison to baseline and to further days of extinction (methamphetamine [53], ketamine [54]). Similarly, we observed increased activity within some saline pretreated rats during the relapse-like behavior testing session. During the relapse-like behavior testing, five of the eight saline rats increased active lever-pressing to above 150% of baseline levels (160–475%). The inactive lever-pressing increase was not significant. This suggests that the saline rats after 12 days of abstinence were very much motivated to get rewarded. This is basically in accordance with the current literature, where active lever-pressing was increased on the first day of the methamphetamine extinction period starting after one week withdrawal period in presence of discrete cue (light) [55]. Within the JMV2959-pretreated rats, during relapse-like behavior testing, the active lever-pressing was decreased to an average of 31.7 ± 8.3% of the baseline mean, and inactive lever-pressing did not differ significantly from the baseline mean. Thus, the GHS-R1A antagonism decreased or prevented the relapse-like/drug-seeking behavior that was observed in saline rats after an abstinence period from methamphetamine, which insinuates an anti-craving effect. To our knowledge, the self-administration method has been used for the testing of ghrelin antagonism only in alcohol and sucrose abuse models in mice and/or rats, where GHS-R1A antagonists JMV2959 and/or (d-Lys3)-GHRP6 reduced intake, preference, and operant self-administration [56,57,58], and in the rat heroin model, where (d-Lys3)-GHRP6 did not influence heroin IVSA under FR1 [59], psychostimulant IVSA models have not been employed so far.

In our IVSA and CPP studies, after pretreatment with GHS-R1A antagonist, we have observed a significant reduction in the methamphetamine rewarding and reinforcing properties. The involved neurobiological mechanisms have to be further evaluated and specified. Based on actual knowledge, we presume that the GHS-R1A antagonist diminution of accumbens dopamine increase induced by amphetamine derivatives plays a crucial role [20], considering that nucleus accumbens shell dopamine is especially involved in strengthening context-drug associations [34]. However, more complex mechanisms should be taken into consideration. The involvement of endocannabinoid system in the accumbens (meth)amphetamine reinforcing processes has been documented; CB1 antagonist attenuated methamphetamine IVSA as well as intra-accumbens methamphetamine self-administration and reduced amphetamine-induced dopamine release specifically in the NAC [60,61,62,63,64]. Recently, interesting interactions among the ghrelin, endocannabinoid, and dopamine systems have been described within the NAC, when pretreatment with CB1 antagonist attenuated ghrelin-induced mesolimbic dopamine release in mice [65]. Also, the GABA-ergic system plays, through multiple pathways, an important role in amphetamine-type stimulant use disorders [66]. Further interactions were found among ghrelin, endocannabinoid, GABA, and opioid systems in the NAC and the VTA, which might play roles in drug reinforcing properties. Namely, pretreatment with JMV2959 in rats significantly reduced/reversed the accumbens *N*-arachidonoylethanolamine (anandamide, AEA) increase and reduced the GABA-increase, both induced by administration of an opioid, and both being considered to participate in opioid reinforcement [46]. Thus, several neural systems, possibly within several brain structures, might cooperate or participate in the observed ghrelin antagonistic JMV2959 effects, including modulated dopamine mechanisms and/or dopamine-independent processes, so further research is necessary. The distinctive constitutive activity of the GHS-R1A may also contribute in the observed effects [67]. The occurrence of GHS-R1A high intrinsic ligand-independent activity indicates that the use of the inverse agonist might be advantageous for clinical practice [68,69]. The substance JMV2959, a widely accepted standard experimental GHS-R1A antagonist, did not reach clinical research yet. So far, from a range of different substances with ghrelin antagonistic effects, only one GHS-R1A inverse agonist, substance PF-5190457, has complied with the strict requirements and has been recently approved for a clinical study in alcoholics [11,70]. However, the potential use of ghrelin antagonism for the treatment of methamphetamine addiction, which was confirmed in our study, can be considered as a useful novel mechanism/approach, which could also be applied to other appropriate clinically acceptable substances with GHS-R1A antagonistic effects.

Ghrelin (acylated) itself shows a rather complex spectrum of effects on systemic metabolism, such as stimulation of gut motility, gastric acid secretion, regulation of glucose metabolism, inhibition of insulin secretion, increase of adiposity etc. Beside its role in reward seeking behavior, ghrelin modulates sleep, stress, anxiety, learning and memory performance, but also protection against muscle atrophy, improvement of some cardiocvascular functions, and role in retinopathy were described (for references see review [71]). Studies with various GHS-R1A antagonists/inverse agonists reflected the participation of more complex mechanisms indicating assumed involvement of different receptor subtypes or various coupled G-proteins etc. [68]. In our study, JMV2959 was administered in single, three, or eight daily doses, always together with methamphetamine or following methamphetamine treatment, which complicated the monitoring of anthropometric and metabolic changes induced by JMV2959. Within our IVSA study we observed no impact of the three daily doses of JMV2959 on body mass of the rats. As expected, due to daily methamphetamine IVSA sessions, the rats did not increase the body mass during the sessions, only on the 12th day of abstinence we observed significant body weight gain in both rat groups, 62 and 63 g in the JMV2959 and saline groups respectively. However, it has been previously described that JMV2959 or other GHS-R1A antagonists abolished several effects of co-infused ghrelin, such as increased weight gain, food intake, and fat mass, suppression of glucose-stimulated insulin secretion or accumbens dopamine increase etc., but the use of the same doses had no impact when administered alone in the absence of ghrelin [20,47,72,73]. On the other hand, chronic JMV2959 did not completely abolish chronic ghrelin-increased food-intake, food efficiency, and increased lean mass (in the contrary to fat mass) in rats, and also some ghrelin effects on hypothalamic gene expression etc. [73]. Thus, participation of other receptor subtypes or des-acyl ghrelin were suggested to explain these behavioral differences. Further research is necessary in order to develop new ligands that selectively target individual signaling pathways linked with GHS-R1A that could treat particular disorders (addiction, obesity etc.) with minimal side effects [68].

Our presented results demonstrated that ghrelin antagonism reduced methamphetamine self-administration, relapse-like behavior, as well as context/place–methamphetamine-associative learning in rats, which strongly encourages further investigation of GHS-R1A antagonists/inverse agonists as potential new pharmacological approach for treatment of methamphetamine addiction.

## 4. Materials and Methods

### 4.1. Animals

Male adult Wistar rats (Velaz, Anlab, Prague, Czech Republic) were used in groups of 8–9 (CPP) and 9–12 (IVSA) animals, weighing approximately 200–250 g during the CPP and 200–320 g during the IVSA experiments. At least seven days before the beginning of the experiments and between the experiments, the rats were given free access to food and water and they were individually housed in polycarbonate cages (IVSA) or three in each box (CPP) with constant humidity (50–60%), room temperature (22–24 °C) and 12 h light/dark cycle (6 a.m.–6 p.m.). The light/dark cycle was reversed in the IVSA experiment (lights on at 6 p.m.), and these rats were handled daily. Procedures involving animals, along with animal care, were conducted in accordance with international laws; protocols complied with the Guidelines of the European Union Council (86/609/EU, 24 November 1986) and the EU Directive (2010/63/EU, 22 September 2010), and followed the instructions of the National Committee for the Care and Use of Laboratory Animals. Experiments were approved by the Expert Committee for Protection of Experimental Animals of the Third Faculty of Medicine, Charles University in Prague, and they were performed in accordance with the Animal Protection Act of the Czech Republic (No. 246/1992 Sb, 15 April 1992); the Protocol permission code MSMT-3778/2016-3 (11 February 2016).

### 4.2. Drugs and Chemicals

Methamphetamine hydrochloride was purchased from Sigma Aldrich (St. Louis, MO, USA). The previously proven GHS-R1A antagonist [74], substance JMV2959 (1,2,4-triazole derivate), was provided by Anton Bespalov, AbbVie, Ludwigshafen/Rhein, Germany. Both substances were dissolved in saline and saline was used as a placebo/control. Methamphetamine (2 or 5 mg/kg) was administered intraperitoneally (i.p.) in volumes of 0.1 mL/100 g of body weight. The selected doses of JMV2959 (1, 3, and 6 mg/kg) were determined based on our previous studies in Wistar rats [46,47,75,76] and the literature [18,20]. The chosen JMV2959 doses had no significant effect on the rat locomotor behavior [47]. JMV2959 was administered i.p. at 0.1 mL/100 g of body weight and always 20 min prior to IVSA or CPP testing, or together with methamphetamine during the conditioning process during one CPP experiment. All reagents were of analytical grade.

### 4.3. Conditioned Place Preference

The biased conditioned place preference (CPP) method, based on our previous experiences and the literature [20,47,77] was performed in rats using three separate experiments and two different experimental designs. We used a three-compartment chamber with the CPP apparatus, with distinct visual and tactile cues in the outer compartments. One outer compartment had wide horizontal black- and white-striped walls and a coarse grid floor, while the other had much finer grid floor and narrow vertical black and white striped walls. The central compartment had no special characteristics, and the gates between the compartments could be opened to allow an animal to move freely between them. All compartments were illuminated by 45 lux. The procedure consisted of pre-conditioning (day 1), conditioning (days 2–9), and post-conditioning (day 10). On day 1 (pre-conditioning), each rat was i.p. injected with saline 20 min prior to testing, then placed in the central compartment with both gates open, and initial place preference was determined during the 20 min in order to determine the spontaneous “least preferred” compartment for each rat. Conditioning was done using a repetitive procedure in which methamphetamine (2 or 5 mg/kg i.p.) was paired to the least preferred compartment. It has been proven that the application of the vehicle/saline does not induce any CPP conditioning. It has been described that JMV2959 has no effect per se on CPP [44]. Therefore these experiments were not included.

### 4.4. Antagonism of Methamphetamine-CPP Expression

In the first experimental arrangement, during the conditioning period, each rat received a total of two i.p. injections per day in a balanced design; methamphetamine (2 or 5 mg/kg i.p.) was administered in the morning and saline conditioning in the afternoon, or conversely in the opposite way. After drug injection, the rat was placed in the appropriate outer compartment (for 40 min, with the gate closed). On day 10 (post-conditioning test session), the rats were placed in the central compartment (with the gates open) and were given free access to both compartments for 20 min. To evaluate the effects of GHS-R1A antagonist on the expression of induced methamphetamine CPP/craving, each rat was acutely injected with JMV2959 (1 or 3 or 6 mg/kg i.p.) or saline (i.p.) 20 min prior to the test session.

### 4.5. Antagonism of the Methamphetamine-CPP Conditioning Process

In the second experimental arrangement, in a separate further study when the effects of GHS-R1A antagonism on the development of methamphetamine CPP were tested, JMV2959 (3 or 6 mg/kg i.p.) or saline (i.p.) was administered repeatedly during the conditioning phase, together with methamphetamine (5 mg/kg i.p.) in separate injections into different sites on the rat.

CPP was always calculated as the difference in the percentage of the total time spent in the methamphetamine-paired (i.e., least preferred) compartment during the post-conditioning and pre-conditioning sessions.

### 4.6. Intravenous Self-Administration

26 naïve male rats were used in the study; groups of 12 (JMV2959) and 8 (saline group) were used in the statistical analyses; two rats were rejected for leaking, and four rats did not reach the minimal stable daily methamphetamine intake. Under ketamine–xylazine anesthesia (ketamine 100 mg/kg i.p., Narketan, Vetoquinol; xylazine 10 mg/kg i.p., Xylapan, Vetoquinol), rats were implanted with a permanent intracardiac silastic catheter through the external jugular vein to the right atrium. The outer part of the catheter exited the skin in the midscapular area and it was fixed in the needleless input (SAI Infusion Technologies, Lake Villa, IL, USA). Animals were controlled daily, and catheters were flushed with heparine (heparine sodium/Heparin Leciva, Zentiva); antibiotics (cefazoline/Cefazolin, Sandoz, Austria) and analgesics (meloxicam, Metacam, Boehringer Ingelheim/Rhein, Germany) were administered for five days. On the sixth day, self-administration sessions were started. The catheters were flushed with 0.3 mL saline, 0.2 mL heparine solution (5 IU) was used to prevent occlusion in the catheters, and blood was aspired to assess the catheter’s patency before each self-administration session. Changes in general behavior, catheter patency, and the body weight of each animal were recorded daily. Experimental cages with two levers located on one side of the cage were programmed by Graphic State Notation 3.0.3. Software (Coulbourn Instruments, Whitehall, PA, USA) and the IVSA sessions were conducted under the fixed ratio (FR) schedule of reinforcement FR1 (each correct response reinforced) until the animal had fulfilled the following conditions for at least seven consecutive sessions in accordance with the literature [48,78]. An active lever-pressing (combined with a cue light) led to the activation of the infusion pump and administration of a single infusion of methamphetamine followed by a 15 s time-out, while an inactive lever-pressing was recorded but not rewarded. The cue light was flashing during dose infusion and off during the time-out. The house light was also flashing, during each infusion. Sessions lasting 180 min were performed twice daily (once daily for each animal), methamphetamine dose was 0.09 mg/kg/infusion/0.1 mL. After a stabile drug consumption for at least seven days (above 70% preference of the active lever, minimum 12 infusions during a session) and after three consequent days with a maximal deviation of 10% rats were pretreated with JMV2959 (3 mg/kg i.p.) or saline (0.1 mL/100 g body weight i.p.) 20 min before IVSA session for three consecutive days. The next day started period of 10–12 days abstinence. During the abstinence period, animals were housed individually in their home cages. On the 10th–12th day of abstinence, the rats were placed again into their IVSA cages for one session, not connected with the pump, to test the tendency for “relapse behavior” (the lever-pressing was monitored). Twenty minutes before this “relapse” session, the rats were again pretreated with JMV2959 (3 mg/kg) or saline (0.1 mg/100 g). The numbers of active and inactive lever-pressing, number of infusions, and methamphetamine consumption (mg/kg) were statistically analyzed. For the last three sessions/days with a stabilized methamphetamine IVSA intake before pretreatment, three consequent JMV2959/saline pretreatment sessions and relapse-behavior sessions were finally used in the statistical analysis.

During the whole IVSA experiment, the body mass of rats was daily monitored, and the difference between groups and possible impact of JMV2959 treatment on the body mass was statistically evaluated within the last eight days before pretreatment, during the three days of pretreatment, the tested relapse-like behavior day and during all evaluated periods (8 baselines + 3 pretreatment days + relapse-like behavior day = 12 days).

### 4.7. Statistical Analysis

Place preference scores (CPP) were calculated as the difference in the percentage (%) of the total time spent in the methamphetamine-paired (i.e., least preferred) compartment during the post-conditioning and the pre-conditioning session. The differences between groups in the CPP were evaluated by a one-way ANOVA, followed by Holm–Shidak post-hoc test. Within the IVSA procedure, a comparison of active and inactive lever pressing was first conducted using a paired sample *t*-test within all analyzed baseline data (last three sessions prior to pretreatments). Statistical differences between the appropriate treatment groups (saline versus JMV2959 pretreatment) relative to the time/session and procedure-related changes of a two-way repeated measures analysis of variance (ANOVA RM) was used with the group (saline versus JMV2959) and session/procedure (3 baseline mean, 1–3 pretreatment) as factors, followed by a Bonferroni-corrected linear contrasts test. General effects of JMV2959/saline administration (data of 1–3 JMV/sal sessions) on methamphetamine IVSA (data of 5–7 baseline sessions = last three baselines before pretreatment) were evaluated by two-way ANOVA with Bonferroni post-hoc test (factors: pretreatment = JMV/saline and procedures = baseline/pretreatment). The difference between groups in the lever-pressing during the relapse-like behavior testing session (“relapse behav”), when the rats were not connected with the infusion pumps, was analyzed separately using the Kruskal–Vallis ANOVA followed by Dunn’s post hoc test. With exception of the relapse-behavior session with no infusions, in each daily session all IVSA parameters were calculated as total number of active and inactive lever-pressing, number of infusions and methamphetamine consumption (mg/kg) during the appropriate 180 min daily session. The representative basal methamphetamine intravenous self-administration (“baseline”) was calculated as a mean of the last three sessions/days prior to the pretreatments, and this mean baseline was used for statistical analyses in the two-way ANOVA RM. Following baseline sessions, in the three subsequent sessions/days saline or 3 mg/kg JMV2959 were administered 20 min before session (“pretreatment, JMV2959/saline”). The “relapse-like behavior” was monitored and calculated on the 12th day of abstinence as the number of active and inactive lever pressings. The changes in the body masses of the rats during the IVSA study were evaluated using two-way ANOVA RM. All statistical tests were evaluated at a significance level of 0.05 (*p* values of <0.05, <0.01 and <0.001 defined statistical significance). All results are presented as the group mean ± SEM.

## Figures and Tables

**Table 1:** Changes of rat body mass within the IVSA study. The body mass changes are represented as group means (JMV2959 group *N* = 12, Saline group *N* = 8) during the chosen periods of the experiment as follows: mean of the last eight days before pretreatment (Bas mean), mean of the three pretreatment days (JMV/sal mean), the day of the relapse-like behavior test (Rel behav), mean of 8 baseline days + 3 pretreatment days + day of relapse-behavior testing (Total mean). Means of the rat groups are presented ± SEM. The statistical significances are described below the table.

Changes of Rat Body Mass within the IVSA Study-Group Means (g ± SEM)				
Group/interval	Bas mean	JMV/sal mean	Rel behav	Total mean
JMV2959 group	286.7 ± 3.3	289.1 ± 6.3	351.0 ± 12.6	292.3 ± 7.3
Saline group	293.2 ± 5.3	298.1 ± 6.1	361.4 ± 18.4	300.1 ± 6.0

Two-way ANOVA RM difference between JMV2959 vs. saline groups: F1,18 = 0.43, *p* = 0.519 (n.s.), effect of time: F11,198 = 33.45, *p* < 0.001 (difference of relapse-like behavior in both JMV2959 and saline groups vs. other parameters), time × group interaction: F11,198 = 0.17, *p* = 0.1 (n.s.); Baseline = mean of 8 days, JMV/sal = mean of 3 pretreatment days, Relapse-like behavior = 1 day, Total mean = 8 Bas + 3 JMV/sal + Rel behav = 12 days; JMV2959 group *N* = 12, Saline group *N* = 8.

## Abbreviations

IVSA	intravenous self-administration
CPP	conditioned place preference
NAC	nucleus accumbens
VTA	ventral tegmental area
